# Sandbox University: Estimating Influence of Institutional Action

**DOI:** 10.1371/journal.pone.0103261

**Published:** 2014-07-23

**Authors:** Jonas Forsman, Richard P. Mann, Cedric Linder, Maartje van den Bogaard

**Affiliations:** 1 Department of Physics and Astronomy, Uppsala University, Uppsala, Sweden; 2 Department of Mathematics, Uppsala University, Uppsala, Sweden; 3 Institute for Futures Studies, Stockholm, Sweden; 4 Department of Physics, University of the Western Cape, Cape Town, South Africa; 5 Faculty of Technology, Policy and Management, Delft University, Delft, The Netherlands; University of Westminster, United Kingdom

## Abstract

The approach presented in this article represents a generalizable and adaptable methodology for identifying complex interactions in educational systems and for investigating how manipulation of these systems may affect educational outcomes of interest. Multilayer Minimum Spanning Tree and Monte-Carlo methods are used. A virtual Sandbox University is created in order to facilitate effective identification of successful and stable initiatives within higher education, which can affect students' credits and student retention – something that has been lacking up until now. The results highlight the importance of teacher feedback and teacher-student rapport, which is congruent with current educational findings, illustrating the methodology's potential to provide a new basis for further empirical studies of issues in higher education from a complex systems perspective.

## Introduction

Interest in modelling higher education as a complex system has grown rapidly during the last decades. Although relevant across the whole spectrum of higher education, the fields of physics, mathematics, and engineering are presently in the forefront of research in this area [1]–[5]. Thus far, this research has mainly taken a theoretical approach to educational issues in higher education. There are only a few exceptions where empirical processes have been analysed from a complex systems point of view, for example, the conceptual understanding of physics [6], physics students' affective learning [7], learning-for-teaching in mathematics [8], and student retention in physics and closely related engineering [9].

Previous research into higher education as a complex system lacks, as Sabelli *et al.* [10] suggest, a system simulation methodology through which researchers and practitioners can pose “what if” questions. These simulations should take into account the nonlinear feedback and interaction effects that are present in higher educational systems [4], [10], where multiple parts of the system adapt to the suggested implementation. Further, these models should be constructed somewhere between the general and the localized so that they can be compared, but also be useful to the local context studied [10].

There are generally two ways of constructing a skeleton for system simulations of processes within higher education; one theoretical, and the other empirical. The approach proposed by Sabelli *et al.* [10] represents an attempt to construct a skeleton from a theoretical basis. A problem with such work is that simulations of such a system will result in the outcomes of the theoretical skeleton being limited by the skeleton itself – the conclusions drawn are only as reliable as the assumptions made in the underlying theory. In this article we present an alternative route to deal with this problem: we demonstrate an empirical path to create a skeleton for the simulation, and propose a framework for performing such “what if”-simulations. As a fruitful way to create such a framework, we propose a generalizable and adaptable methodology in order to identify complex interactions in educational systems. We use Multilayer Minimum Spanning Tree and Monte-Carlo methods to propose a way to explore how manipulation of these systems may be affecting educational outcomes. Additionally, we report on what our simulations suggest are the most important factors for improving educational outcomes.

We have chosen to focus on the credits students achieved, which is an integral part of student retention, as the target of our analysis. This is because a critical first step for students continuing towards graduation is for them to complete their courses, thus getting the credits needed to continue their studies, also called *academic withdrawal* [11]. Internationally, enhancing student graduation rate has received a great deal of attention over the last ten years, especially in science and engineering [12]. However, implemented institutional actions to address the problem have not had the anticipated effect, as evidenced by the unchanging (or even declining) graduation rates in all areas of science, technology, engineering, and mathematics [13].

Researchers, building on central models of student retention - which academic withdrawal is a part of [11], [14]–[15] - have found empirical inconsistencies when predicting student retention. Examples of inconsistencies are the predictive power of age and gender [16], students' goal commitments [17], and financial aid to the students [18]. The emergence of these inconsistencies indicates that even after the identification of many of the critical aspects of students' educational experiences, estimating the effectiveness of proposed changes in institutional practices remains highly problematic. This is probably because most parts of an educational system are interrelated, i.e., are complex [4], [19]–[21]. Consequently, in such an interrelated system, the ability to identify aspects that produce both effective and ineffective changes to educational practice becomes of paramount importance [22].

In an effort to address this challenge, we report on the creation of a virtual ‘Sandbox University’ (SU), where changes in institutional practice can be simulated, estimated, and compared. The SU is empirically estimated based on questionnaire data consisting of first-year study experiences obtained from engineering students who have physics as a part of their curriculum at the highly regarded Technical University of Delft. We do this in order to: 1) create a localized model which can inform local institutional practice; and, 2) create a system in which it is possible to circumvent the problem that proposed changes can be hindered by exogenous processes of the real-world system. For example, the changes forced on the SU will be “noiseless” – that is free of influence from a changing external environment outside the system being studied, which is of course impossible in the real-world system [23]. Thus, our research question is: *how can targets for changes in institutional practice be effectively identified using an empirically-informed Sandbox University?*

## Method and Data

### Dataset

Our Sandbox University is composed from 78 previously identified critical aspects of student retention – aspects of students' experience of studying at a university that have been found to have a positive impact on students' abilities to persist through their higher education studies - which also includes students' credits achieved. The data was collected in three-year bachelor programmes from a wide variety of engineering and engineering science programmes in the fall of 2010 at the Technical University of Delft in the Netherlands. The cohort studied consisted of first-year students and the data collection was carried out by using an online questionnaire. The response rate was 25% (573 of 2292). The questionnaire was designed to obtain students' first-year study experiences [24]–[25]. In total, the questionnaire together with additional data from the central student administration (for example, age, students' credits achieved, etc.) consisted of 78 items. The items, their links (edges), and their justifications where grounded in the reports of contemporary research field. These are given in Appendix S1. The full description of the questionnaire can be found in Appendix S2.

### Ethics section

The University (TU Delft) where the data was collected required no specific ethics submission, had no ethics board in place, and had no formal procedures to be followed in human subjects' research. Even though this was the case, an informal committee of university researchers and administrators was gathered before data collection to approve the design of the study. This committee consisted out of the Director of Student and Teacher Services and two research professors. Moreover, the data collection followed the ethical guidelines as described by Cohen, Manion, and Morrison [26], which meant that informed consent was obtained from the participating students. Full information on the goals of the study, which researchers and administrators were involved and how they could be contacted, and the fact that the information that they provided on the questionnaire would be linked with data from the central student database were all disclosed. However, it was made explicitly clear that both sets of data would only be stored and analysed after any information that could link data to a student had been removed. Participation was voluntary and would not have any effects on their grades. The participants agreed to the terms of research by entering their unique student ID which made it possible to link the questionnaire answers to the university's student database. Students who did not agree to these terms, or who did not complete the questionnaire in full, were not included the data base and none of their information was saved. Any information which could be used to identify individual students was removed before any analysis on the data was undertaken. All items included in the questionnaire were strongly grounded in previously published peer-reviewed research (see Appendix S1).

### Workflow

**Figure 1:**
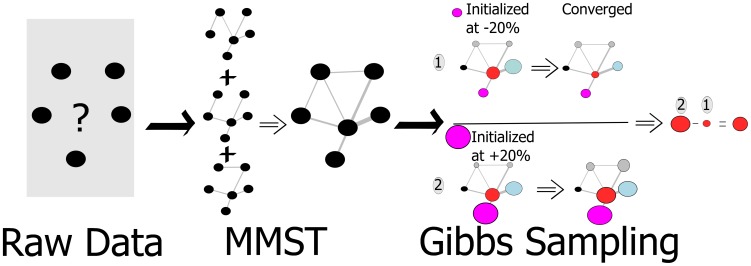
Workflow of the proposed methodology. The magenta node is where effect of changes is sought. The black nodes are nodes which are held constant. The blue and grey nodes represent First- and Second-order nodes as per the grouping in Table 2.The red nodes are the target which is to be estimated.

Figure 1 illustrates the methodology workflow chosen in order to create a simulation of a Sandbox University. To establish a network structure, a minimum spanning tree (MMST) analysis [27] was undertaken of the raw data. In order to establish what would happen if changes in the system were introduced, Gibbs sampling was used with two initial starting points. The estimations of changes reported on in the results section is the difference between the estimated values when the Gibbs sampling converged.

### Network estimation

The relationship between the 78 aspects was estimated through an implementation of MMST analysis [27]. There are multiple ways of estimating a network structure from correlated data, for example Correlations [28], Partial correlation estimations [29] and Bayesian Networks [30]. However, if other method of estimation of network structure had been chosen, the proposed methodology still would hold.

**Figure 2:**
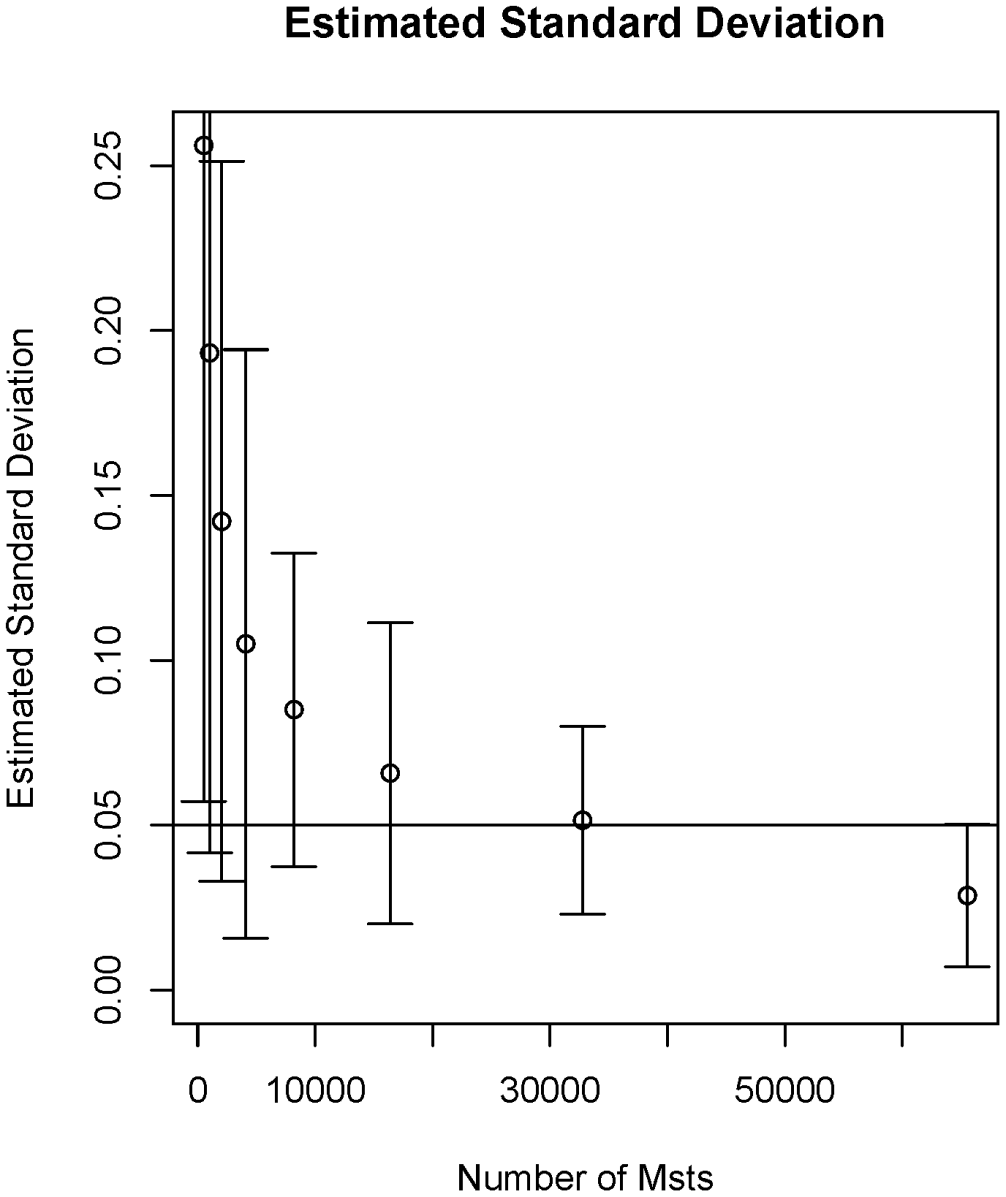
Convergence of MMST creation.

The MMST analysis was chosen because, in contrast to a correlation network where everything tends to be connected to everything else, the edges are not a result of choosing a cut-off of the strength of the correlation but through the reproducibility of edges (as shown in Figure 2). The MMST instead aims to identify the strongest edges; edges which are valid in most subsets of the data, and weak edges; for example a correlation which is only valid and present in a few subsets, which correlation analysis sometimes can miss when analysing the full dataset. Therefore MMST estimation favours edges which are always, rather than sporadically, present in the system. Furthermore, the MMST method is well established for network estimation (e.g. [27]) and is straightforward to implement.

In this study, we used an implementation of MMST analysis [27] that was made in the statistical environment **r** [31]. The methodology bootstraps [32] the data and a minimum spanning tree (MST) is created for each subset which corresponds to the strongest significant Spearman correlations [33]. The MMST is created by the union of each MST created. The number of MSTs making up the MMST was increased until the difference, including one standard deviation, between two MMSTs created by the same number of MSTs was below 5% error in each edge as shown in Figure 2.

Edge weights (strength of links) in the MMST represent the frequency of that correlation found in each bootstrapped sample. In our implementation, both positive and negative correlations were present in the MST and thus positive and negative relations within the network were identified and colour coded in the visualization as grey (for positive relationship) and red (as negative relationships). In the visualization produced, the 15% weakest (non-frequent) edges are removed. Before this manipulation was done almost every node had weak edges to all other nodes, which resulted in a very noisy visualisation.

The elements of the created network are the measured aspects as per the questionnaire. In each iteration of MMST analysis correlations between questionnaire items are calculated for subsets of the raw data, which are, in turn, recalculated to a distance matrix. Then a minimum spanning tree [34] is generated to link all elements using the fewest number of edges and the lowest edge weights (in the minimum spanning tree case, distances) as possible. Over several iterations of the algorithm, different edges are identified. The frequency with which each possible edge is included in the spanning tree determines the strength of connection between two elements. We thus built a network representing the whole system using these frequencies as the weight of edges between every pair of elements. We expect that the strongest edges indicate genuine pairwise connections, whereas weaker edges may indicate relationships mediated by intermediate elements. We therefore prune this network by removing weak edges, retaining the strongest 75% of connections.

### Estimation of influence

In order to estimate the influence and uncertainty that a change in an aspect would have on the target aspect, Gibbs sampling [35] was undertaken in the networked system. This Monte-Carlo methodology iteratively estimates the value of each unfixed node in the network, which is based on the conditional probability distribution of that value with respect to the *current* estimated values of directly adjacent nodes. Over many iterations the values generated for each node converge to the joint posterior probability distribution for those node values, conditioned on the constant values of the fixed nodes. In this way, Gibbs sampling can be used to determine the likely change in one node based on forced changes in another. The target aspect chosen was the number of credits achieved by the students, which we chose as a suitable proxy for academic withdrawal [11], as it corresponds to students having sufficient number of courses to be allowed to continue towards their degree.

For example, in our network, students' previous grade in mathematics, students feeling that they have done sufficient preparatory study, and students' who only want to pass and not care about the grades are adjacent to the number of credits achieved. Following Equation (1) and (2), over the iterations, the value of *credits achieved* are re-estimated based on the re-estimations of the values of students' previous grade in mathematics, student's thinking they study enough, and students' who only want to pass and not care about the grades.

The Gibbs sampling drew from a normal distribution where the mean of this distribution is the weighted mean of the adjacent nodes (Equation 1). 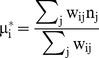
(1)where the estimated mean is 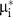; 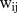 is equal to the edge weight between adjacent aspect i and j; and, 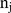 is the value of aspect j.

The standard deviation used for the Gibbs sampling was estimated by the unbiased estimator for the weighted sample variance (Equation 2),
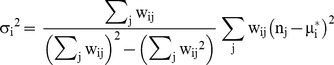
(2)


Where 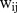 is then the edge weight between aspect i and j; 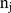 is the value of aspect j; and, 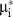 is the estimation of the weighted mean (as per Equation 1). Thus, the standard deviation is low when the adjacent nodes are of similar value, and high when adjacent nodes have values far from each other.

**Figure 3:**
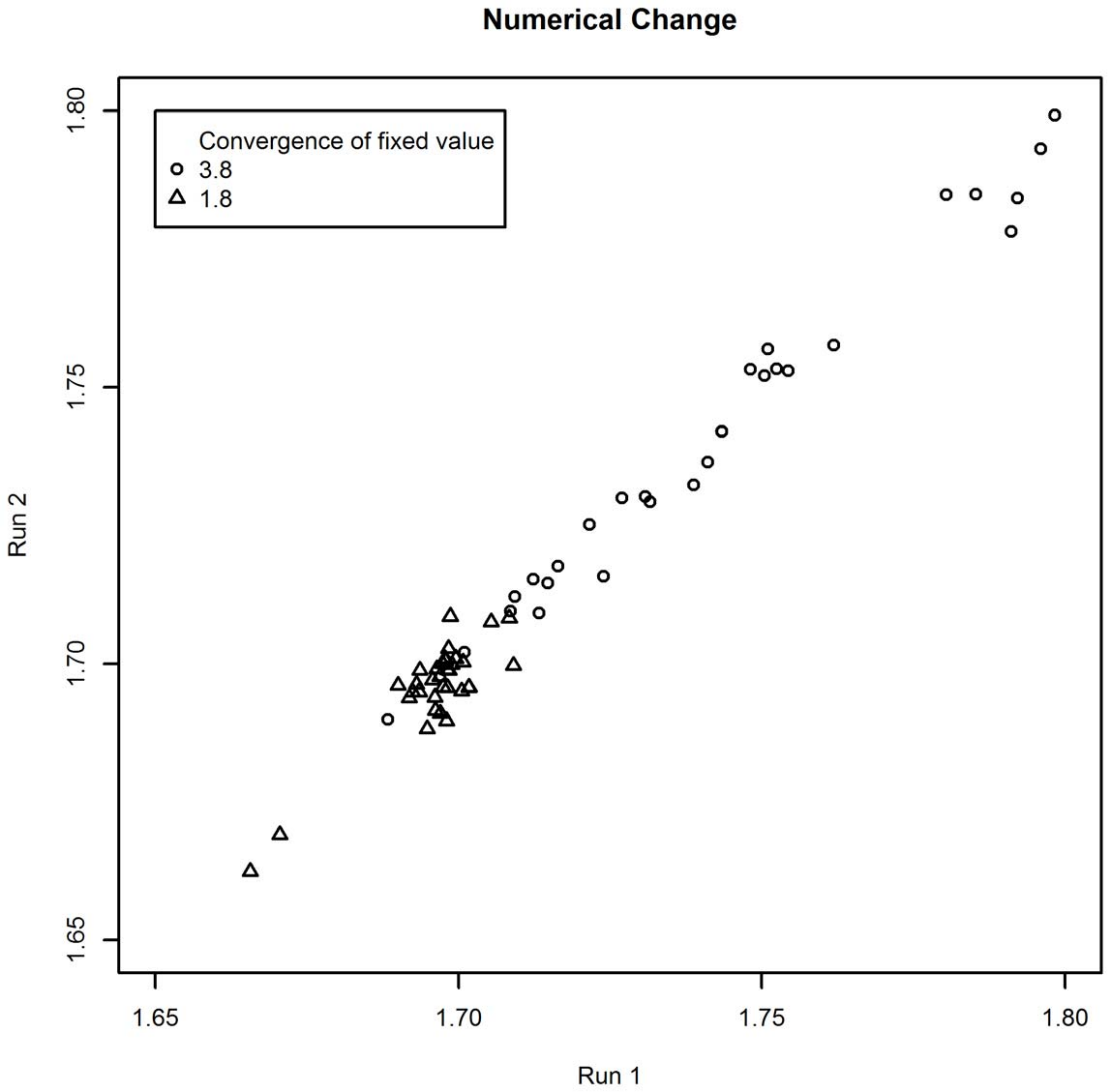
Convergence of the Gibbs sampling for the estimation of the numerical changes.

**Figure 4:**
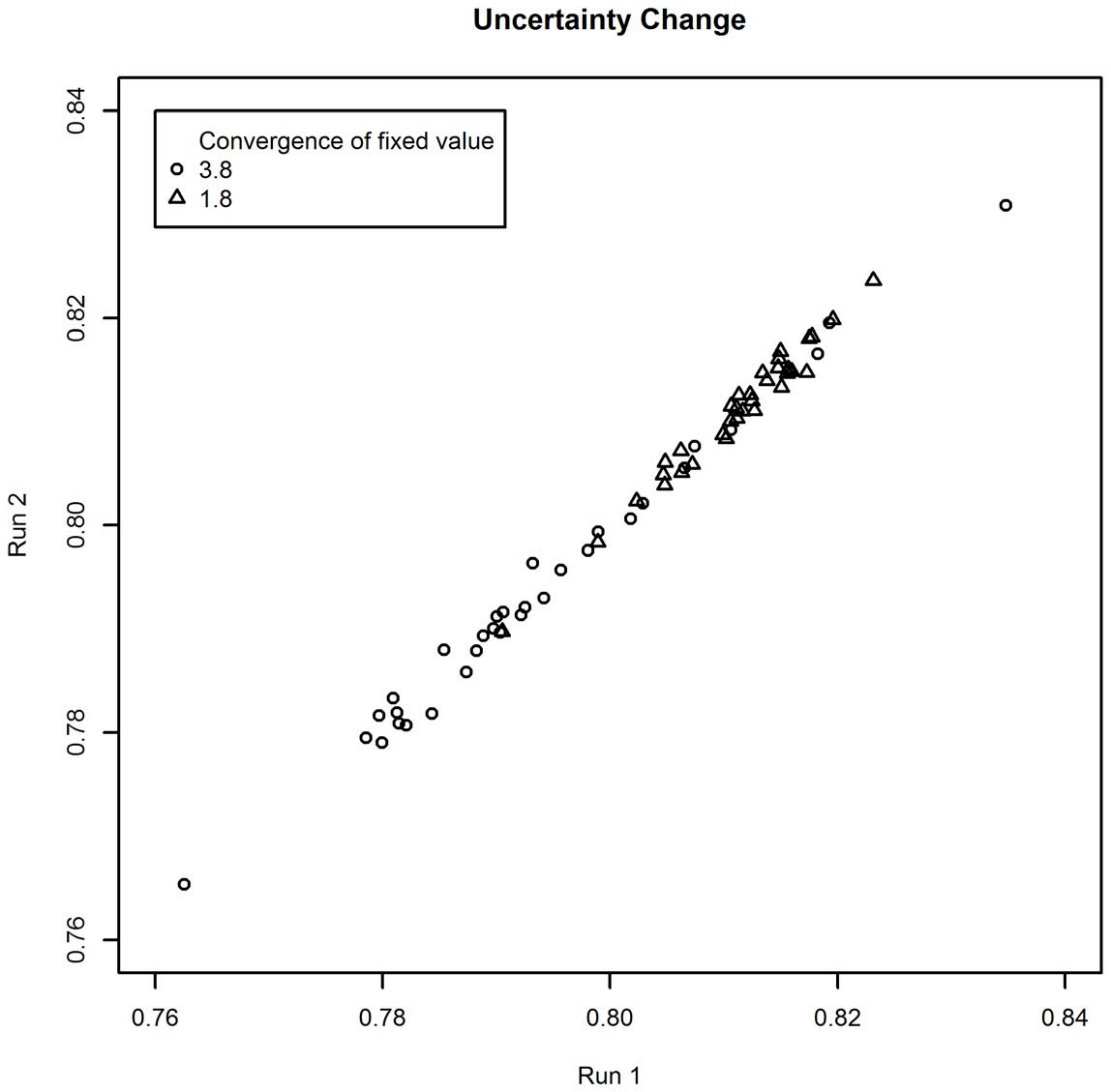
Convergence of the Gibbs sampling for estimation of the standard deviation.

Each iteration of Gibbs sampling estimated all interrelated aspects in a random order. The Gibbs sampling ran for 60 000 iterations, with a burn-in period of 1000 to allow for convergence, and with a thinning of 100 to increase the statistical independence of generated values. The estimations are the results of what would have happened to the target aspect when proposing that you could “improve” an aspect from 20% below to 20% above the average of the measured aspect. Figure 3 and Figure 4 show that the sampling converged within these parameters for both changes to the aspects undertaken.

## Results

The SU was estimated from the observed correlations. However, there are multiple ways of building such networks, such as from a theoretical starting point [10]. Using the empirical data as a starting point, this methodology estimates the network relating to students' first year of study experiences and thus creates a SU in a localized context. Our methodology creates a skeleton network though which influence can travel on multiple paths, it also allows feedback structures, thus allowing for non-linearity between different parts of the system.

As not all aspects can be easily changed, the 78 aspects measured by the questionnaire were then divided into three groups (see Table 1): Constant (consistent), First-order variable, and Second-order variable. The constant group is constituted of aspects that cannot be changed in a reasonable time-period, such as parents' education. The First-order variable group is constituted by aspects that are possible to change (within reason), while the Second-order group consists of aspects that can only be changed by changing adjacent aspects. The grouping of aspects is based on current problems in science and engineering education, which are not arguably in the selection procedures of students [36]. It is not a question of declining enrolment in these areas, but a question of the retention of students [37]. As an example, on average, only 50% of students enrolled in a science or engineering program in the United States eventually complete their degrees [14]. Thus, the grouping is focused around what can be changed when the students are already at the university, after the selection process has taken place.

**Table 1:** Three groups of critical aspects.

Constant	First-order	Second-order
Students' age	Teacher expectations (2 Expec)	Students' re-enrolment expectations
Stem profile combination*	University facilities (5 Uf)	Students' experiences of university facilities (2 Ufs)
Students' parents' education	Scheduling (6 N)	Degree importance (2 Important)
Students' biological gender	Course materials (4 Cm)	Language skills (2 Language)
Students' housing situation	Teacher behaviours (7 Tb)	Fraternity membership
Students' impairments	Travel time to campus	Students' experience of course materials (2 Cms)
Students' exposure to university PR	Assessment and feedback (9 Af)	Students' study behaviour (20 Sb)
Students' prior education		Students' self-evaluated skills (3 Skill)
Previous achievement in mathematics		
Previous achievement in physics		

Note: The number beside each group of aspects indicates how many aspects are measured in each grouping, and the abbreviation after indicates what those are in the Appendix S1.

*See Appendix S1: item B_Ment_profile for more information.

**Figure 5:**
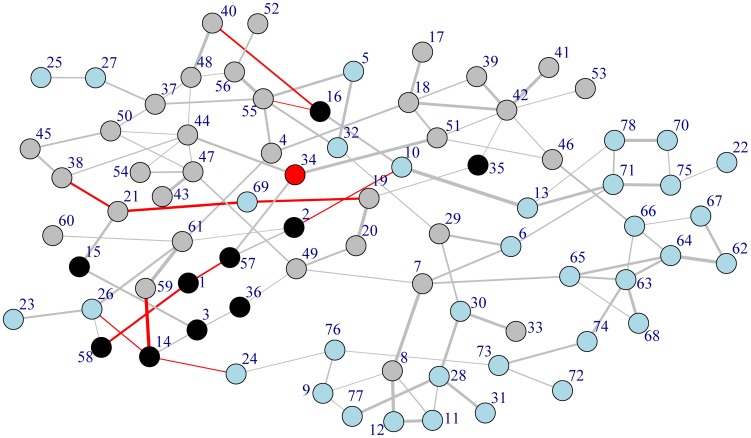
Visualization of estimated interrelationships. Black nodes are the constant nodes, blue are the First-order grouped nodes and grey are the Second-order grouped nodes, the red node is the target node for the proposed changes to institutional practice. The widths of the edges indicate the strength of the estimated links, and the colour represents positive (grey) and negative (red) relationships.

The relationships, as estimated by MMST analysis, between these aspects resulted in a network map of how the aspects interrelate (see Figure 5).

In order to estimate influence and uncertainty of a change in one aspect on the target aspect, Gibbs sampling [35] was undertaken in the networked system. This Monte-Carlo methodology estimates the conditional probability for unknown values of nodes in the network based on values of adjacent nodes, and can therefore be used to determine the likely change in one node based on forced changes in another. The target aspect chosen was the number of credits achieved by the students, which acts as a proxy for academic withdrawal [11].

**Figure 6:**
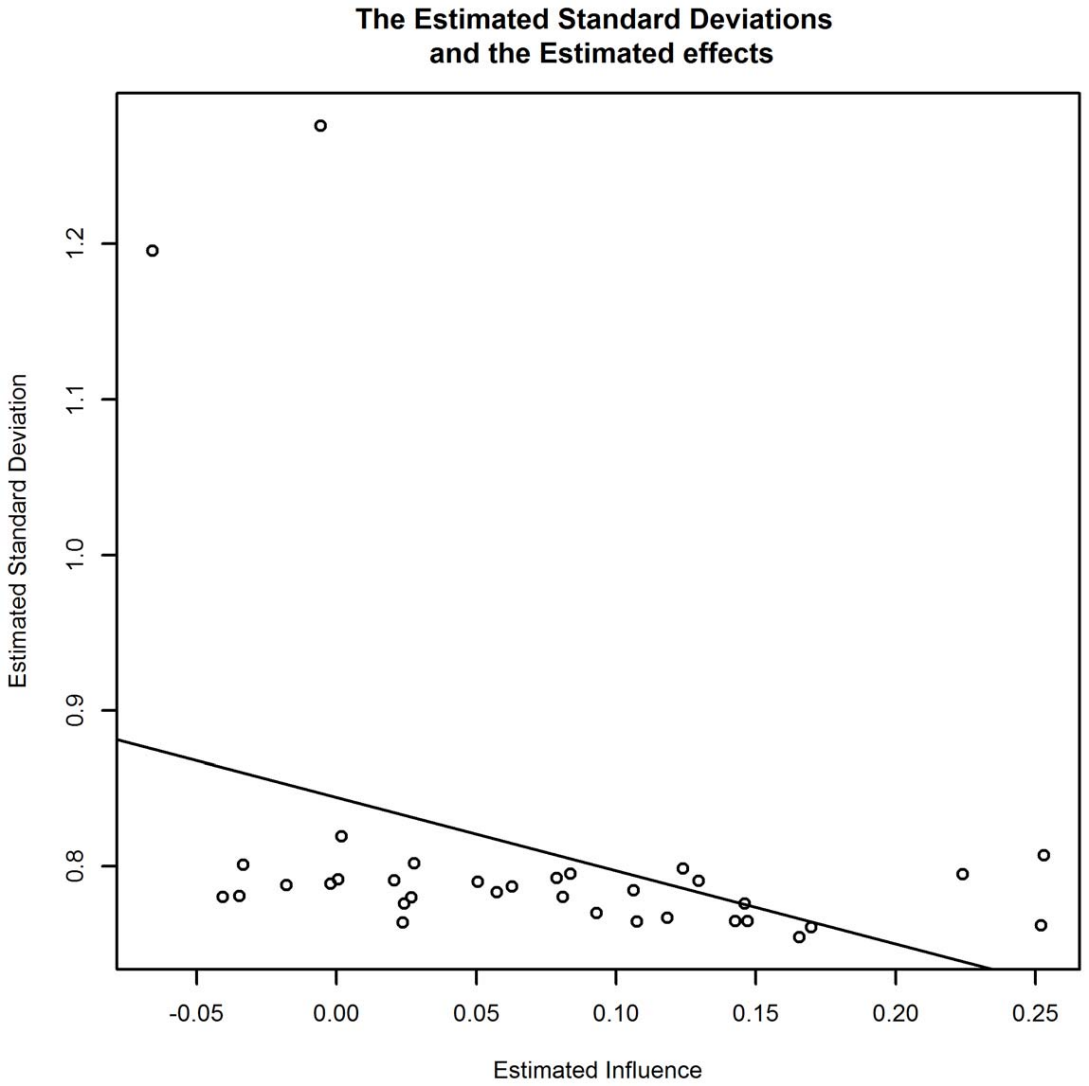
Shows that the uncertainty tends to be slightly higher if the estimated influence of a particular aspect is higher.

The resulting estimations were compared with the estimated standard deviation of each aspect (shown in Figure 6). These can be interpreted in terms of the following: targets that show greater potential for bringing positive change tend to have a larger span of possible resulting effects.

The estimated change in student credits achieved is compared in Table 2 with Hattie's synthesis [38] of over 800 meta-analyses consisting of more than 5000 studies. The comparison is not made in relation to a ‘gold standard’, but rather in a tentative way to make it visible how themes found in the analysis correspond to well established findings. The study undertaken by Hattie collated the effect sizes of different meta-studies of influences relating to learning outcomes and ranked these from highly positive, to highly negative in relation to the mean effect size found. A ranking of 1 to 138 of analysed effects was estimated by Hattie, where the top 40 (those well above the mean effect size) are those effects that were deemed worthwhile [38].

**Table 2:** Results from the Gibbs Sampling.

First-order Aspects	Estimated Change (%)	Estimated Standard Deviation (%)	Hattie Rank	Hattie Theme
(5) Teacher expectations - Expec_difficulties	11	30	10	Teacher - Feedback
(32) Course materials - Cm_material	9	32	-	
(64) Teacher behaviours - Tb_empathize	8	30	11	Teacher - Teacher-Student Relationships
(63) Teacher behaviours - Tb_content	8	30	11	Teacher - Teacher-Student Relationships
(30) Course materials - Cm_feedback	8	30	10	Teacher - Feedback
(31) Course materials - Cm_late	7	30	10	Teacher - Feedback
(65) Teacher behaviours - Tb_enthusiasm	6	29	11	Teacher - Teacher-Student Relationships
(66) Teacher behaviours - Tb_explain	6	30	11	Teacher - Teacher-Student Relationships
(74) Assessment & feedback - Af_level	6	30	10	Teacher - Feedback
(71) Assessment & feedback - Af_constr	6	30	10	Teacher - Feedback
(62) Teacher behaviours - Tb_available	5	30	11	Teacher - Teacher-Student Relationships
(6) Teacher expectations - Expec_interest	5	28	10	Teacher - Feedback -
(25) Scheduling - N_lectures*	5	80	-	-

Note: Only aspects where effect sizes which have a >5% mean positive estimated effect on students' credits achieved are shown. The number before the First-order aspect provides a visual link to the variables in Appendix S1 and Figure 5.

*Highly unstable.

Table 2 shows that the influential aspects estimated are comparable to Hattie's high ranked effect sizes of influence of the synthesis of student achievement [38]. Of note is the high variance of the effect estimation of the number of lectures (labelled (25) N_lectures). This suggests that it is possible for the estimated effect to have a very high positive, or even a substantial negative, influence on credits achieved, but not in a consistent way (resulting effects).

The largest estimated effect comes from improving teachers' ability to deal with students' expectations, which relates to students' experience of teachers' feedback on how students are doing with the courses. Teacher feedback (especially dealing with students' expectations) has long been recognised as an important factor for student learning within the field of educational research [39]. From our network estimations, the most likely effects of improving teachers' ability to deal with students' expectations would be that it would positively affect students' study behaviour (particularly dealing with the experienced pace of study in a course). The main connections to students' credits achieved are students' study behaviours.

Other aspects showed lower estimated effects and are thus not reported here. This is because from this simplified model it is highly uncertain that these would have any desirable effects on credits achieved. However, lower estimations could, when introducing more complexity, have more substantial effects, but not consistent ones.

## Discussion

We built a virtual Sandbox University by using empirical data from student questionnaires to identify aspects of the student experience that are most strongly linked. These links were then used to construct a network of interrelated aspects. Based on this network we simulated the effect of changing aspects of the student experience that can plausibly be directly manipulated, investigating the expected impact of each such intervention on student credits achieved. We thus identified the areas where interventions would be most likely to substantially improve student outcomes – such as students' credits achieved, and student retention.

The limitations described previously when using a theoretically driven skeleton for simulations are mirrored in this study, as our results are only as good as the methodology used for creating the network. However, our methodology can be used as an exemplar of how such skeleton networks can be fruitfully estimated. The network created also only covers first-year engineering students. How the network might change over time is beyond the scope of this article.

Our simulation resulted in two important broad and common themes: Teacher feedback and Teacher-student relationships, which have been found to be at the top end of effectiveness when their impact on student achievements has been studied [38]. This, together with the fact that our findings are also congruent with findings from the student retention literature [11], [14]–[15], [40], suggests that the methodology has validity for the context studied.

Within the resulting common themes, an unexpected finding is that the aspect corresponding to students obtaining, and being informed about, the required materials for the courses ((32) Cm_material) has a mean effect size above 5%. This is surprising since this has neither been recognized as the top influence on student credits achieved [38] nor is this highlighted in student retention research [11], [14]–[15], [40]. Following Sabelli *et al.* [10] we argue that this is one of the strengths of our methodology since the influence of this aspect can be attributed to the local context. Moreover, there is an important point to be made here. In our simulations we found a highly unstable aspect; the number of scheduled lectures. Clearly, interventions targeting such unstable aspects may produce conflicting outcomes. We argue that this could provide an explanation for the kind of conflicting results currently found in student retention research [16]–[18].

The approach presented in this article represents a generalizable and adaptable methodology for identifying complex interactions in educational systems and for investigating how manipulation of these systems may affect outcomes of interest. This approach enables the effective identification of successful and stable initiatives within higher education that can affect students' credits achieved and student retention – something that has been lacking up until now [10]. The focus in our article has been on networks created from empirical data, but clearly similar approaches could equally be applied in theoretically derived networks. Evaluating the likely effectiveness of interventions in this way will lead to more effective management of educational environments, which, in turn, will generate more stable outcomes in such environments.

## Supporting Information

Appendix S1 **Full description of variables in the study.**
(DOCX)

Appendix S2 **Full description of questionnaire used in the study.**
(DOCX)
