# Correction: Sandbox University: Estimating Influence of Institutional Action

**DOI:** 10.1371/journal.pone.0141705

**Published:** 2015-10-23

**Authors:** Jonas Forsman, Richard P. Mann, Cedric Linder, Maartje van den Bogaard

A reference is omitted from the figure caption for Fig 2. Please see the complete, correct Fig 2 caption here.

**Fig 2:**
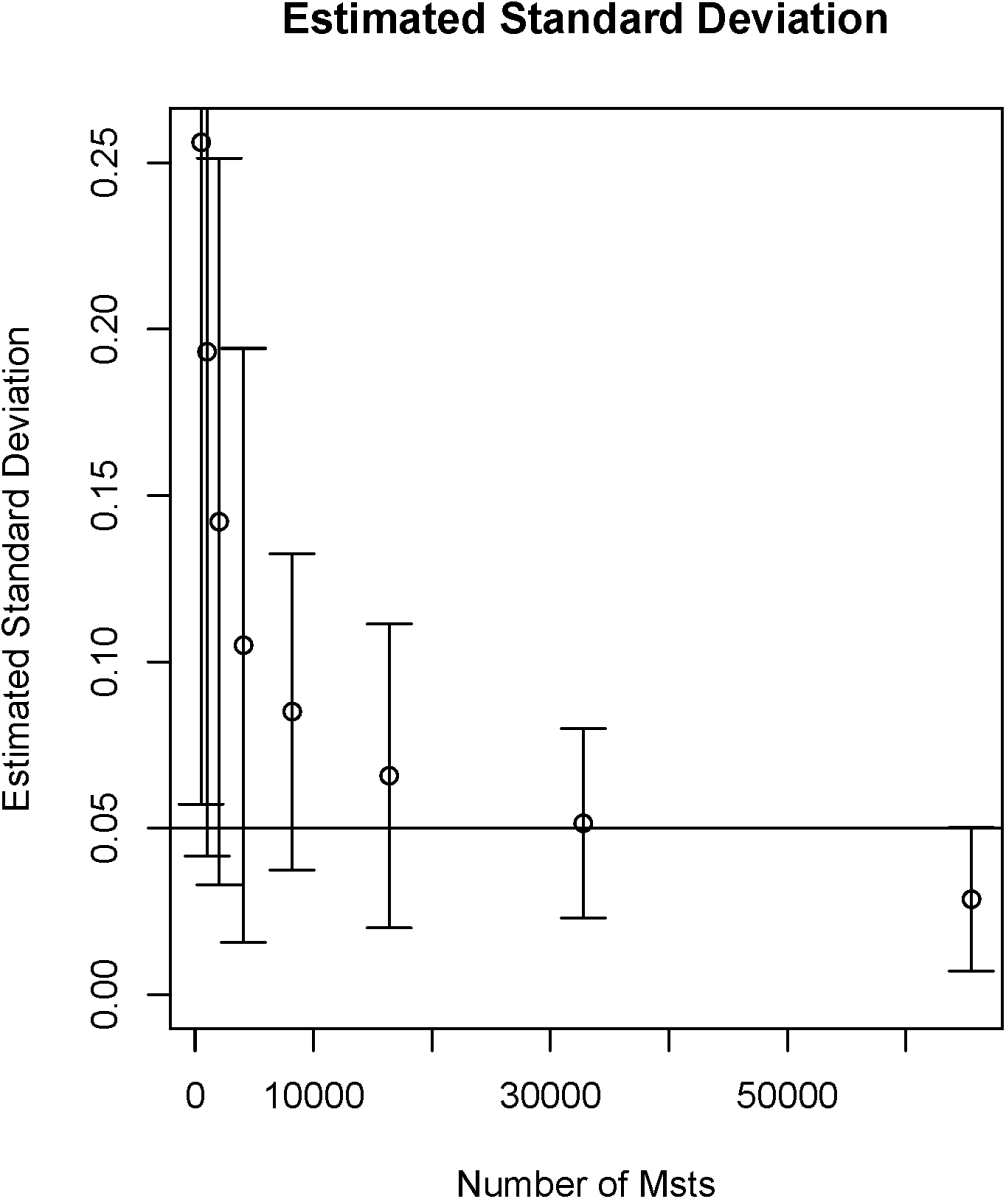
Convergence of MMST creation. (see also [41])

The reference is: Forsman J, Van den Bogaard M, Linder C, Fraser D (accepted for publication 2014). Considering student retention as a complex system: a possible way forward for enhancing student retention. European Journal of Engineering Education.
