# Supplementary material for: Sandbox University: Estimating Influence of Institutional Action
Source: PLoS One. 2014 Jul 23;9(7):e103261. doi: 10.1371/journal.pone.0103261 (PMC4108410; doi:10.1371/journal.pone.0103261)
Supplement: Appendix S1 — Full description of variables in the study. (DOCX) [file pone.0103261.s001.docx]

# Appendix 1

TABLE 1: List of items included and their link to previous research

| No. | Name | Description | From Reference |
| --- | --- | --- | --- |
| 1 | Age | Age on first day of the first academic year of enrolment | 1-5. |
| 14 | Gender | Students’ biological gender | 2, 3, 6-14 |
| 57 | SE_math | Secondary Education grade math | 1, 2, 4, 5, 10, 13-16 |
| 58 | SE_physics | Secondary Education grade physics | 2, 4, 5 |
| 36 | Prior_ed | Secondary Education profile or other prior education | 5, 13, 17 |
| 19 | Language_Dutch | Self rating on 5 point scale: Mastery of language | 2, 4, 17, 18 |
| 20 | Language_Eng | Self rating on 5 point scale: Mastery of language | 17; 18; 2; 10; 4; 13; 19. |
| 60 | Skill_math | Self rating on 5 point scale: math skills. | 5, 8, 18-2 |
| 61 | Skill_physics | Self rating on 5 point scale: physics skills | 8, 18. |
| 59 | Skill_comp | Self rating on 5 point scale: computer skills | 8, 17; 18. |
| 69 | Traveltime_tot | Commute to campus per week in hours | 22 |
| 27 | N_travel_days | N days travel to campus | 22 |
| 3 | Ed_parents | Do parents have a degree in Higher Education | 1, 2, 3, 5, 7, 12-14, 17, 23, 24 |
| 2 | B_Ment_profile | STEM profile combination of profiles based on Beta-mentality model. | 14, 15, 17, 19, 25 |
| 21 | Member_frat | Membership of student fraternity | 22, 26; 27. |
| 16 | Imp_N_Total | Number of physical or learning impairments (14 predefined impairments + 2 open). | 15, 17 |
| 15 | Housing_sit | Housing situation | 16, 15, 17 |
| 5 | Expec_diff | Does difficulty programme match expectations. Rating on 5 point scale. | 2, 12, 17, 28 |
| 4 | Expec_BSA | Do you expect a positive re-enrollment advice (half of credits of 1st year)? Rating on 5 point scale. | 5, 12, 29, 30 |
| 6 | Expec_interest | Do you find your studies as interesting as you expected? Rating on 5 point scale. | 11, 17, 28, 29 |
| 17 | Important_Delft | Importance of studying /obtaining a degree in Delft. Rating on 5 point scale. | 12; 17; 29; 30 |
| 18 | Important_P | Importance of obtaining P diploma in 1st year. Rating on 5 point scale. | 12, 17, 28, 29 |
| 35 | PR_total | Number of PR related activities students have participated in. | 9, 17 |
| Items teachers [Tb], assessment [Af] and facilities [Uf] were on a 5 point scale. Items on curriculum organizations [Cm] were on a 4 point scale. | | | |
| 63 | Tb_content | Teacher has plenty of content knowledge. | 1, 2, 5, 12 |
| 66 | Tb_explain | Teachers can explain concepts in different ways. | 1, 2, 5, 12, 17 |
| 67 | Tb_hall | Teachers take time to answers questions from students in the lecture hall. | 1, 2, 5, 12, 28 |
| 68 | Tb_master | Teachers have truly mastered the subjects they teach. | 1, 5, 12 |
| 62 | Tb_available | Teachers are available when students have questions. | 1, 2, 5, 8, 17, 28 |
| 64 | Tb_empathize | Teachers can empathize with the students. | 1, 5, 8, 12, 17, 28 |
| 65 | Tb_enthusiasm | Teachers are enthusiastic about their courses. | 1, 5, 8, 12, 17 |
| 75 | Af_proj | In projects expectations are clear. | 17, 22 |
| 71 | Af_constr | Feedback on project is constructive | 17, 22 |
| 70 | Af_consist | Feedback on project is consistent | 22 |
| 78 | Af_trans | Final feedback on project is transparent. | 17, 22 |
| 72 | Af_exp | In exams expectations are clear. | 17, 22, 28 |
| 73 | Af_feedback | Feedback on formative tests was constructive. | 17, 22 |
| 74 | Af_level | Level of exams was representative of course level. | 22 |
| 76 | Af_repres | Content of exams was representative of course. | 22 |
| 77 | Af_time | Enough time to sit the exam. | 22 |
| 12 | Uf_studyF | There are plenty of working spaces to study quietly at the faculty. | 17, 22 |
| 11 | Uf_studyC | There are plenty of working spaces to study quietly on the campus. | 17, 22. |
| 9 | Uf_stmen | Student mentor was accessible. | 17, 22 |
| 13 | Uf_tcmen | Teacher mentor was accessible. | 12, 22; 17. |
| 10 | Uf_studsup | Study support is accessible. | 12, 17, 22 |
| 7 | Ufs_atm | There is a good atmosphere at the faculty. | 12, 15, 17, 22 |
| 8 | Ufs_relax | There is plenty of space to relax at the faculty. | 17, 22 |
| 32 | Cm_material | Materials were difficult to understand. | 17, 22 |
| 31 | Cm_late | Materials were not available or too late. | 17, 22, 28 |
| 28 | Cm_book | It was difficult to find out what books/materials we needed to study. | 17, 22 |
| 30 | Cm_feedback | I received insufficient feedback. | 17, 22 |
| 33 | Cms_spread | Course load was spread unevenly. | 2, 17, 22, 28 |
| 29 | Cms_courses | The courses did not appeal to me. | 17, 22 |
| Indicators of Student behavior are included in a large number of studies, for example [17, 27, 31]. The items below are based on [32] and [33]. | | | |
| 47 | Sb_goal | I set goals and stick to them. |  |
| 54 | Sb_syst | I don’t study systematically. |  |
| 55 | Sb_tempo | The pace of the programme is too high for me. |  |
| 41 | Sb_deepl1 | When I study I truly want to understand it. |  |
| 45 | Sb_exam | I mainly study for the test. |  |
| 37 | Sb_behind | I’m always behind on my work. |  |
| 38 | Sb_bursts | I work in bursts. |  |
| 43 | Sb_eff | I work out strategies to spend my time efficiently. |  |
| 48 | Sb_hard | I fail to work hard enough. |  |
| 44 | Sb_enough | I don’t do enough for my studies. |  |
| 52 | Sb_pause | I interrupt myself often and take breaks. |  |
| 50 | Sb_keepup | I keep up with my work. |  |
| 56 | Sb_toomuch | I have too much going on. |  |
| 40 | Sb_concen | I can concentrate well, even if I find a subject tough. |  |
| 49 | Sb_help | If I can’t work it out myself, I find help. |  |
| 46 | Sb_forget | After exams I forget everything again quickly. |  |
| 51 | Sb_mark | I want to pass the test, I don’t care for the mark. |  |
| 42 | Sb_deepl2 | When I wrap up a course, I want to feel like I really learned something. |  |
| 39 | Sb_check | If I fail a test, I go in to check my mistakes. |  |
| 53 | Sb_prep | When I fail an exam, I will prepare in a different way next time. |  |
| 23 | N_courses | Number of courses scheduled in one education period | 2-4 |
| 24 | N_exams | Number of exams scheduled in one education period. | 2-4 |
| 22 | N_active | Number of scheduled hours for active teaching formats, such as projects and practicals. | 2-4 |
| 25 | N_lectures | Number of scheduled hours of lectures. | 2-4 |
| 26 | N_mandatory | Number of scheduled hours where presence is mandatory. | 22 |
| 34 | P_EC_N | Number of credits obtained in first year. |  |

References

1. Bruinsma M, Jansen EPWA (2007) Educational productivity in higher education: an examination of part of the Walberg Educational Productivity Model. School Effectiveness and School Improvement 18: 45 – 65.
2. Jansen EPWA, Bruinsma M (2005) Explaining achievement in higher education. Educational Research and Evaluation 11: 235 - 252.
3. Van den Berg MN, Hofman WHA (2005) Student success in university education: a multi-measurement study of the impact of student and faculty factors on study progress. Higher Education 50: 413 – 446.
4. Van der Hulst M, Jansen E (2002) Effects of curriculum organisation on study progress in engineering studies. Higher Education 43: 489 – 506.
5. Prins JBA (1997) *Studieuitval in het wetenschappelijk onderwijs. Studentkenmerken en opleidingskenmerken als verklaring voor studieutval* [Student drop out from university. Student and curricula factors as explanations for drop out]. Njimegen: Nijmegen University Press.
6. Araque F, Roldán C, Salguero A (2009) Factors influencing university drop out rates. Computers & Education 53: 563 – 574.
7. Georg W (2009) Individual and institutional factors in the tendency to drop out of higher education: a multilevel analysis using data from the Konstanz Student Survey. Studies in Higher Education 34: 647 – 661.
8. Vogt CM (2008) Faculty as a critical juncture in student retention and performance in engineering programs. Journal of Engineering Education 97: 27 – 36.
9. French BF, Immekus JC, Oakes W (2005) An examination of indicators of engineering students’ success and persistence. Journal of Engineering Education 94: 419 – 425.
10. Zhang G, Anderson TJ, Ohland MW, Thorndyke BR (2004) Identifying factors influencing engineering student graduation: a longitudinal and cross-institutional study. Journal of Engineering Education 93: 313 - 320.
11. Beekhoven S, De Jong U, Van Hout H (2002) Explaining academic progress via combining concepts of integration theory and rational choice theory. Research in Higher Education 43: 577 – 600.
12. Beekhoven S, De Jong U, Van Hout H (2003) Different courses, different students, same results ? An examination of differences in study progress of students in different courses. Higher Education 46: 37 – 59.
13. Moller-Wong C, Eide A (1997) An engineering student retention study. Journal of Engineering Education 86: 7 – 16.
14. Felder RM, Mohr PH, Dietz EJ, Baker-Ward L (1994) A longitudinal study of engineering student performance and retention II. Rural/urban student differences. Journal of Engineering Education 83: 209 - 218.
15. Beekhoven S, De Jong U, Van Hout H (2004) The impact of first-year students’ living situation in the integration process and progress. Educational Studies 30: 277 - 290 (2004).
16. Oseguera L, Rhee SB (2009) The influence of institutional retention climates on student persistence to degree completion: A multilevel approach. Journal of the Association for Institutional Research 50: 546 - 569.
17. Warps J, Wartenbergh F, Hogeling L, Pass J, Kurver B, Muskens M (2010), *Een goede start in bètatechniek. Studiekeuze, studiesucces en studieuitval in hoger bètatechnisch onderwijs* [A good start in engineering. Choice of study, study success and drop out in higher engineering education]. Njimgen: Platform Bèta Techniek Research.
18. Burtner J (2005) The use of discriminant analysis to investigate the influence of non-cognitive factors on engineering school persistence. Journal of Engineering Education 94: 335 – 338.
19. Besterfield-Sacre M, Atman CJ, Shuman LJ, Characteristics of freshman engineering students: models for determining Student Attrition in Engineering. Journal of Engineering Education 86: 139 - 150.
20. Veenstra CP, Dey EL, Herrin GD (2008) Is modeling of freshman engineering success different from modeling of non-engineering success? Journal of Engineering Education 97: 467 - 479.
21. Jansen EPWA, Bruinsma M (2005) Explaining achievement in higher education. Educational Research and Evaluation 11: 235 - 252.
22. Van den Bogaard MED (2011) A qualitative inquiry into first year engineering student success. In W. Hernandez (Ed.), Research in Engineering Education Symposium 2011, 70, Madrid.
23. Felder R, Felder GN (1998) A longitudinal study of engineering student performance and retention. V. Comparisons with traditionally-taught students. Journal of Engineering Education 87: 469 - 480.
24. Felder RM, Forrest KD, Baker-Ward L, Dietz EJ, Mohr PH (1993) A longitudinal study of engineering student performance and retention: I. Success and failure in the introductory course. Journal of Engineering Education 82: 15 - 21.
25. Woodcock A, Graziano WG, Branch SE, Habashi MM, Ngambeki I, Evangelou D (2012) Person and thing orientations: psychological correlates and predictive utility. Social Psychological and Personality Science 4: 116 – 123.
26. Berger JB, Milem JF (1999) The role of student involvement and perceptions of integration in a causal model of student persistence. Research in Higher Education 40: 641 - 664.
27. T. H. Delft (1958)*, Mislukking en vertraging van de studie; verslag van een onderzoek 1953-1957* [Failure and delay in the study: report of research 1953-1957].Delft: TH Delft, Delft.
28. Need A, De Jong U (2001) Do local study environments matter? A multilevel analysis of the educational careers of first-year university students. Higher Education in Europe 26: 263 – 278.
29. Thomas LS (2000) Ties that bind: A social network approach to understanding student integration and persistence. Journal of Higher Education 51: 591 - 615.
30. Cabrera AF, Castaneda MB, Nora A (1992) The convergence between two theories of college persistence. The Journal of Higher Education 63: 143 - 164.
31. Seymour E, Hewitt NM (1997) Talking about leaving: Why undergraduates leave the sciences. Boulder: Westview Press.
32. Zimmerman BJ Kitsantas A (2007) The hidden dimension of personal competence: self-regulated learning and practice. In A. J. Elliot & C. S. Dweck (Eds.), Handbook of competence and motivation. New York: The Guilford Press p. 509.
33. Schmitz B, Wiese B (2006) New perspectives for the evaluation of training sessions in self-regulated learning: Time-series analyses of diary data. Contemporary Educational Psychology 31: 64 – 96.
