# Supplementary material for: Sandbox University: Estimating Influence of Institutional Action
Source: PLoS One. 2014 Jul 23;9(7):e103261. doi: 10.1371/journal.pone.0103261 (PMC4108410; doi:10.1371/journal.pone.0103261)
Supplement: Appendix S2 — Full description of questionnaire used in the study. (DOCX) [file pone.0103261.s002.docx]

**Survey First year engineering students (coh 2010)**

Dear student,

Student success is currently receiving a lot of attention in media and policy as it affects everyone at Delft University of Technology.

As you probably know, not all the students finish their first year or their courses. We want to find out where the bottlenecks in the courses and curriculum organisation are, so we can start to improve on those. Therefore we need your help.

In this research we ask all first year students of all the courses in Delft to fill out this survey. This survey is slightly different from the surveys in which you are asked about your opinion on the subjects you took. The questions in this survey do not just deal with education, but also with your personal situation. Another difference between this survey and the regular education surveys is that in this survey we intend to link the outcomes with your data in the Osiris central student database. We do this to find out what factors relating to education matter for student progress. This is important for us to understand where to start with our efforts to improve.

We will treat all your information with great care, we store the information in a safe place and when we report on this research, data and findings will not be retraceable your person. The data will only be used for this research.

By filling out this survey you give us permission to link the outcomes of this survey to your information in Osiris. Filling out the survey will take 10 to 15 minutes.

This research is executed by the Office of Education and Student Affairs together with researchers from the faculty of Technology, Policy and Management. If you have any questions regarding this research, please contact m.e.d.vandenbogaard@tudelft.nl.

Thank you.

Maartje van den Bogaard

Researcher TPM

On behalf of Director of Student and Teacher Services at the Office of Education and Student Affairs.

1. This survey is only available in Dutch. If you do not speak Dutch, please click here.

I do not speak Dutch.

2. Please fill out your 7-digit student identification number using the drop down menus.

**Prior education**

3. Based on what qualification did you enrol in Delft University of Technology?

- Pre university education diploma, focus on natural sciences and technology.

- Pre university education diploma, focus on natural sciences and health.

- Pre university education diploma, focus on natural science and technology and health

- Entrance exam of DUT

- First year diploma of university of applied sciences.

- Other, namely……

4. Did you take extra elective courses in pre university education?

- No

- Yes, namely……

5. Is this your first course in tertiary education?

- No (skip to question 8)

- Yes

6. You indicated that you were enrolled in a different course before. Which course? ……

7. Did you finish this course (did you obtain for example a B.A., B.Sc., M.A., etc)?

- No

- Yes

8 What grades did you obtain for mathematics and physics in your exams in secondary school?

If you are not sure, give an estimation.

Math 1 2 3 4 5 6 7 8 9 10

Physics 1 2 3 4 5 6 7 8 9 10

9. Have you ever been retained in school?

- No

- Yes

**Skills**

10. Which languages do you speak and what is your mastery level of these languages?

Dutch poor mediocre fair good excellent not applicable

English poor mediocre fair good excellent not applicable

11. How do you assess your own skills in these areas:

Math poor mediocre fair good excellent

Physics poor mediocre fair good excellent

Computer skills poor mediocre fair good excellent

(such as matlab)

**Housing situation**

12. Where do you live right now?

- With my parents/guardians (skip to question 16)

- Housing with private facilities (skip to question 16)

- With a landlord/lady (skip to question 16)

- Housing with shared facilities (student room)

- Other, namely……. (skip to question 16)

13. You indicated that you live in housing with shared facilities. How many flatmates do you have?

Number of flatmates:

14. Is your house associated with a student union (fraternity/sorority)?

- No

- Yes

15. Are any of your flatmates enrolled in the same course you take?

- No

- Yes

**Commuting**

16. What is your average commuting time for a one way trip from your home address to your faculty?

Please indicate the commuting time in hours and minutes.

If your trip takes less than an hour, fill in ‘0’ in the hours box.

Hours: Minutes

17. How many days a week did you come to the faculty during this educational period?

Average number of travel days:

**Unions and associations**

18. Are you a member of a student union?

(multiple answers possible)

- Yes, of a student union in Delft

- Yes, of a sports association in Delft

- Yes, of a sports association close to my parents

- Yes, of a cultural association in Delft

- Yes, of a cultural association close of my parents

- Yes, of my study association

- No, I am not a member of any association

- Other, I am a member of another association or club, namely: ……

**Parental level of education**

19. Did your parents/guardians graduate from a course in tertiary education (university or university of applied science)?

- One of my parents/guardians

- Both my parents/guardians

- Neither of my parents/guardians

- I do not know.

20. Are there attributes that may limit your ability to study, such a learning difficulties or physical impairments?

- No (skip to question 22)

- Yes

21. You indicated that you have some attributes that may limit your ability to study. Can you indicate what these attributes are?

(multiple answers possible)

- Dyslexia

- (Protracted) Pain

- Limitations regarding moving

- Limitations regarding seeing

- Limitations regarding hearing

- Limitations regarding speaking

- Limitations regarding stamina

- Chronic fatigue

- Concentration problems

- Sleep disorders

- Depression or mood swings

- Fear or panic attacks

- Condition in the autistic spectrum

- Other, namely

22. Indicate with on the statements below is most applicable to you, which one less and which one the least.

1= most applicable, 2= less applicable, 3= least applicable

- I want to know how things work and how they are assembled. I am good at the sciences and find the study materials interesting. I am not sure what sort of job I would like to have, but it has to fit with what I like to do and what I am good at.

- I enjoy technical gadgets, but I do not feel like tinkering with them when they break down. I found the subjects on the natural sciences boring and old fashioned. In the future I would like to make a lot of money and hold a position with a high status.

- I believe that technology can contribute to developments in society. I think about my future and I will look for a job that allows me to contribute meaningfully to society and that allows me to work with people and work on my personal development.

**Expectations**

23. Is the level of difficulty of the course as you expected?

- Much more difficult

- More difficult

- As difficult as expected

- Easier

- Much easier

24. Is the course as interesting as you expected?

- Much less interesting

- Less interesting

- As interesting as expected

- More interesting

- Much more interesting

25. Do you expect to obtain a pass to the second year (there is academic dismissal when students fail to obtain less than 30 out of 60 credits in their first year of studying)

- No

- Probably not

- I do not know

- Probably

- Yes

26. How important is it for you to study at DUT/ to graduate form DUT?

- Very unimportant

- Unimportant

- Neutral

- Important

- Very important

27. How important is it for you to obtain your propaedeutical (first year) diploma in one year?

- Very unimportant

- Unimportant

- Neutral

- Important

- Very important

**Information on DUT and courses**

28. Which sources of information did you use when you were deciding on which course you would enrol in?

(choose between 1 and 5 answers)

- General campus visits to the institute/ courses

- Intensive campus visits, such as masterclasses or student-for-a-day events

- Student mentors through “Beta 1 on 1” at my secondary school

- Campus visit with my secondary school

- Information event from DUT on my secondary school

- Information from my parents and/or peers, such as siblings and friends

- I requested information from DUT/ course myself by telephone or e-mail

- Visit to the ‘StudieBeurs” event in Utrecht

- Brochures and other printed information materials from DUT/ courses

- Printed materials from independent sources such as Keuzegids Hoger Onderwijs, Elsevier’s ‘De Beste Studies” rankings, etc.

- YouTube channel of DUT and/or www.itunesu.tudelft.nl

- Websites of institutes and/or comparison websites such as [www.studiekeuze123.nl](http://www.studiekeuze123.nl)

- None of these sources of information

- Other sources, namely……..

**Reasons for choosing Delft University of Technology**

29. Can you indicate if the reasons stated below played a role for you

- prior to your studies (while you were making a choice regarding your field of study)

- now, while you are studying

- after your studies (when you think of the future)

A reason can be valid at different moment in your life, therefore you can tick multiple boxes for the same reason. It is possible that a stated reason does not apply to you, in that case you do not tick any of the boxes for that stated reason.

Prior Now After

DUT has a good reputation

I feel attracted to the city of Delft and its student life

Short and/or easy commute

The campus atmosphere

The faculty atmosphere

I can relate to the students who are enrolled in the course

I can relate to the teachers in the course

The entrance requirements of the course

The level of difficulty of the course

The content of the curriculum and subjects

The balance between time for education and time for relaxation

The course’s focus on theory

The course’s focus on practice

The curriculum offers a lot of study opportunities during and after the course

The curriculum fits well with my capacities

I find the curriculum’s contents appealing.

The support that is offered in the curriculum

The possibilities to find a interesting job

The possibilities to find a well paid job

The possibilities to find a high status job

The possibilities to find a job offering many opportunities

**Teacher expertise**

32. Below you find statements that have to do with how you perceive the quality of the teachers you have had in the previous educational period.

We want to get an impression of your perceptions. It is possible that one or two teachers stood out, either in a positive or negative way. If that is the case, you can elaborate in the box below the question.

5-point Likert scale: -/-= not applicable at all, +/+=completely applicable.

The teachers can convey and explain the teaching materials well.

The teachers can explain difficult concepts in different ways.

The teachers take time to answer questions from the students during lectures.

The teachers truly master the contents, they really know what they talk about.

The teachers are available when I have questions.

The teachers can relate to the students well.

The teachers are enthusiastic about their subjects.

33. Space to explain or clarify your answers.

**Assessment and feedback**

34. We want to find out more about how you perceived the assessment in your subjects. Assessment refers to interim feedback on your project or on partial exams, such as COZ. We are also interested in your opinions on the final exams. Below you find statements that have to do with these aspects of assessment. You can elaborate on your answers in the box below the statements.

5-point Likert scale: -/-= completely disagree, +/+=completely agree, NA= not applicable

In the projects the expectations are clear.

The interim feedback on your project was constructive (e.g. afterwards you had a clear conception of what needed to be improved upon)

The interim feedback on your project was consistent (e.g. the teacher held on to the same standards and criteria)

The final assessment of the project was transparent (e.g. you understood what the assessment and grade were based on)

In the theoretical subjects the expectations at the exam were clear.

The feedback on the partial exams was constructive (e.g. afterwards you understood what you had been doing wrong)

The level of difficulty of the exams was a reflection of the difficulty of the subjects.

The questions in the exams were a reflection of the contents of the subjects.

There was enough time to answer the questions in the exams.

35. Space to explain or clarify your answers.

**Facilities**

36. Below you find statements regarding the facilities that are available to you. Please indicate to what extent you agree with the statements. You can clarify your answers in the box below the statements.

5-point Likert scale: -/-= completely disagree, +/+=completely agree, NA= not applicable

There is a positive and stimulating atmosphere in the faculty.

There are enough quiet places to study in the faculty.

There are enough quiet places to study on campus.

There are enough places to relax in the faculty.

The student-mentor was accessible for me.

The teacher-mentor was accessible for me.

The student-counsellors are accessible for me.

37. Space to explain or clarify your answers.

**Curriculum organization**

38. Sometimes things go wrong in the daily course of events. As long as that does not hinder you, that is not so bad. Sometimes it gets in your way, for instance when a syllabus is not available in time or when the courseload of a subject is not spread evenly over the education period.

Below you find statements that have to do with the curriculum organization of subjects in the previous period. Please indicate to what extent you experienced hindrance as a results of topics covered in the statements. If you feel the statement does not apply, tick the N.A. box. You can clarify your answers in the box below the statements.

-/-= a lot of hindrance -=hindrance +=a little hindrance +/+= not hindrance NA=not applicable

The course load was unevenly spread over the period.

The teaching materials were difficult to understand.

The teaching materials became available too late or not at all.

It was difficult to find out which books I needed for my subjects.

I received insufficient feedback on my assignments.

The subjects of this period did not appeal to me.

39. Space to explain or clarify your answers.

**Study behaviour and study strategy**

40 Below you find statements regarding your study behaviour and study strategies. Please indicate to what extent they apply to you.

5-point Likert scale: -/-= not applicable at all, +/+=completely applicable.

1. I set personal short-term and long-term goals and stick to them.
2. I do not work systematically.
3. The tempo of the course is too high for me.
4. When I study the teaching materials, I really want to understand them.
5. I mainly study for the test.
6. I am frequently behind on my school work.
7. I work in bursts.
8. I come up with and try different strategies to spend my time on independent study efficiently.
9. I do not succeed in studying hard enough.
10. I do not do enough for my studies.

41. Below you find more statements regarding your study behaviour and study strategies. Please indicate to what extent they apply to you.

1. I regularly interrupt myself to smoke, drink coffee, take a walk, etc.
2. I keep up with the teaching materials as much as possible.
3. I have too much on my mind.
4. I can concentrate well, even when I find the subject matter difficult.
5. When I don’t understand something, I find people who can help me, like the teacher or a flat mate.
6. After the exam I quickly forget what the subject was about.
7. I want to pass the test, I do not care for the grade.
8. When I finish a subject I want to have the feeling I really learned something.
9. If I fail an exam, I check the exam to find out what I did wrong.
10. If I fail an exam, I prepare myself in a different way for the resit.

**Time**

42. Please indicate the number of hours you spent on average per week on the following activities in the past education period.

Submit the average number of hours in whole numbers. If you have not spent any time on an activity, fill in a 0.

Present at the faculty

Independent study at home

Social activity (such as unions, sports, etc)

Paid employment

Gaming

**Follow up**

Thank you for filling out this survey.

Based on the results of this survey we intend to do a follow-u survey on a small number of topics. We intend to do this in February/March 2011.

43. Can we approach you for taking part in this follow up survey? You can still decide at that time whether you participate or not.

- I do not want to be approached.

- I do not object to being approached.

My e-mail address is:

**Finish**

This is the end of this survey. If you have any questions or comments on this research, please contact [m.e.d.vandenbogaard@tudelft.nl](mailto:m.e.d.vandenbogaard@tudelft.nl). Thank you once again for your participation.
